# Glycerol-Enhanced Gum Karaya Hydrogel Films with a Sandwich-like Structure Enriched with Octenidine for Antibacterial Action against Multidrug-Resistant Bacteria

**DOI:** 10.1021/acsomega.5c02915

**Published:** 2025-07-02

**Authors:** Eva Černá, Vilém Neděla, Eva Tihlařiková, Jana Brtníková, Zdenka Fohlerová, Břetislav Lipový, Lukáš Vacek, Filip Růžička, Jana Matulová, Lucy Vojtová

**Affiliations:** † Central European Institute of Technology, 613011 Brno University of Technology, Purkyňova 656/123, 612 00 Brno, Czech Republic; ‡ Institute of Scientific Instruments of the Czech Academy of Sciences, Královopolská 147, 612 00 Brno, Czech Republic; § Faculty of Electrical Engineering and Communication, Brno University of Technology, Technická 10, 616 00 Brno, Czech Republic; ∥ Department of Burns Medicine, Third Faculty of Medicine, Charles University and University Hospital Královské Vinohrady, Škrobárova 1150/50, 100 34 Prague, Czech Republic; ⊥ Department of Microbiology, St. Anne’s University Hospital Brno and Faculty of Medicine, 37748 Masaryk University, Pekařská 53, 602 00 Brno, Czech Republic

## Abstract

This study explores the innovative approach in the development of freeze-dried hydrogel films, leveraging the unique properties of gum Karaya (GK), poly­(vinyl alcohol) (PVA), poly­(ethylene glycol) (PEG), and glycerol with a coating of octenidine dihydrochloride (OCT). These innovative hydrogel films exhibit at a certain glycerol concentration a sandwich-like structure, achieved through a tailored freeze-drying process, which enhances transparency and mechanical stability. OCT provides superior antibacterial performance, effectively combating multidrug-resistant bacteria with a controlled and gradual release mechanism, surpassing conventional OCT solutions that require frequent reapplication for infected wound treatment without the creation of bacterial resistance. Advanced environmental scanning electron microscopy (A-ESEM) reveals the complex microstructure of the hydrogel, highlighting the dense surface layer and interconnected porous bulk. Variations in glycerol concentrations proved to significantly impact hydrogels’ properties. Increasing the glycerol concentration decreases the pore size (around 4.5 μm) while enhancing the polymer network density and flexibility. However, low concentration increases the pore size (7.8–15.6 μm), impacting enhanced swelling behavior and hydrolytic stability. OCT’s rapid antibacterial action, releasing over 30% within the first hour and maintaining prolonged activity for up to 2 weeks, emphasizes the material’s potential for diverse applications. Hydrogels’ remarkable transparency, porosity, structural stability, and antibacterial efficacy against both Gram-positive *Staphylococcus aureus* and Gram-negative *Escherichia coli* strains suggest promising uses as transparent dressings, biomedical devices, and infection-resistant surfaces.

## Graphical abstract

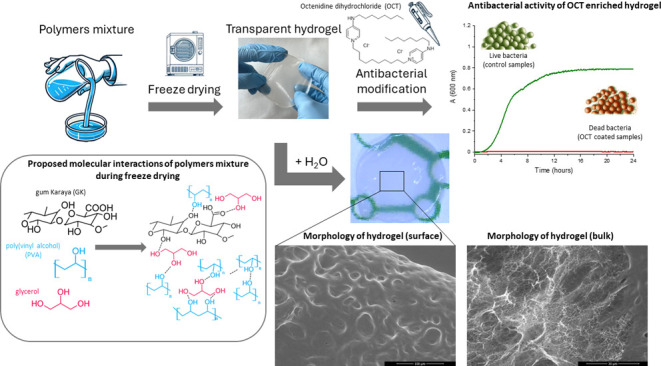

## 1 Introduction

Hydrogels, as versatile and highly tunable materials, have garnered significant attention due to their unique composition–structure–property relationships, which enable their use in advanced applications ranging from biomedical devices to antibacterial coatings.^1,2^ First synthesized by Wichterle and Lim, hydrogels have evolved significantly over the past half-century, becoming essential in various fields due to their tunable structural, mechanical, and biological properties.^3,4^ Characterized by their three-dimensional structures, hydrogels consist of a cross-linked polymer network that is hydrophilic and insoluble in water, allowing them to bind and retain significant water, mimicking the natural extracellular matrix (ECM).^5,6^ Their adaptability and a wide range of fabrication methods allow for fine-tuning their composition, making them suitable for diverse applications, including biomedical devices, coatings, antibacterial surfaces, wound healing, and flexible electronics.^3,7^

Despite extensive research on hydrogels, transparent hydrogels composed of naturally antimicrobial biopolymers like gum Karaya (GK) with synthetic polymers such as poly­(vinyl alcohol) (PVA), in combination with plasticizers poly­(ethylene glycol) (PEG) and glycerol, fabricated using freeze-drying, represent a novel approach that uniquely balances biocompatibility, mechanical stability, and bioactivity. This study explores this innovative combination to overcome limitations in existing hydrogel formulations. The hydrogel composition, fabrication method, and cross-linking mechanism significantly impact the final properties of the hydrogel, such as mechanical stability, swelling, or transparency. Freeze-drying is a fabrication method widely used in the food and pharmaceutical industries to stabilize products, and it can be utilized in hydrogel fabrication. Unlike traditional film casting or freeze–thaw methods, freeze-drying in the first step freezes water, preventing chemical, biochemical, or microbiological processes, then removes water from the hydrogel matrix by sublimating ice, forming a controlled microstructure with interconnected pores.^8,9^ This property is exploited in hydrogel fabrication as it produces materials with uniform pores of a size depending on the hydrogel composition, enhanced swelling capacity, increased surface area, mechanical properties, and ability to easily incorporate bioactive molecules.^10,11^ The addition of plasticizers such as glycerol or PEG increases hydrogel elasticity and transparency, which is essential for transparent coating applications in tissue engineering.^12,13^

Gum Karaya (GK) is an industrially important polysaccharide, highly biocompatible and biodegradable as a natural compound with inherited antimicrobial activity.^14–16^ It is an abundant and relatively inexpensive material derived as a resin from the *Sterculia urens* tree that can form gels and potentially profitable commercial products in combination with other polymers (PVA, chitosan,^17^ silk fibroin^18^). Synthetic polymers, namely, PVA, PEG, poloxamers, poly­(*N*-vinyl-2-pyrrolidone) (PVP), and poly­(acrylic acid) (pAA), are used as wound dressings, as they possess high water absorption and retention, gel formation, and sufficient mechanical strength.^19^

PVA, a highly hydrophilic synthetic biopolymer soluble in water, possesses biocompatibility, biodegradability, and nontoxicity while having excellent film-forming properties due to the hydrogen bonding of −OH groups and creation of crystalline domains resulting in mechanically strong, cross-linked PVA hydrogels.^20,21^ Unfortunately, inadequate elasticity, brittleness, and stiffness are incompatible with its use alone as a hydrogel dressing.^22,23^ PVA can be reduced using plasticizers. Glycerol is a transparent, nontoxic, and viscous liquid widely used in the biomedical field as a plasticizer and cryoprotectant.^24,25^ These properties can be utilized in hydrogel fabrication by using freeze–thaw or freeze-drying methods. Glycerol molecules in a hydrogel matrix influence its microstructure and reduce the creation of crystallinities that affect hydrogel properties, such as increased transparency and elasticity.^20,24,26^ PEG is a hydrophilic polymer widely used in hydrogel fabrication for its ability to form a highly swellable network with chain flexibility and thermal resistance.^27^ It is suitable for use in the biomedical industry for its nontoxic and inert behavior.^28^

Hydrogel bioactivity, namely, antibacterial properties, can be secured in different ways; a hydrogel matrix composed of various chemicals has antibacterial activity on its own, or the hydrogel serves as a drug delivery system by adding antibiotics, antiseptics, antibacterial proteins/peptides, or nanoparticles.^29,30^ These bioactive modifications are essential nowadays due to increased multidrug resistance against conventional antibiotics since they represent a global problem and threaten the healthcare system, influencing millions of lives.^31,32^ This work secures antibacterial activity by integrating antiseptic octenidine dihydrochloride (OCT), enhancing antimicrobial performance to treat potential or present infection. OCT has a wide antimicrobial spectrum and is highly effective at lower concentrations (0.1%) in a short time while covering many bacterial or fungal strains (including multidrug-resistant strains), e.g., methicillin-resistant *Staphylococcus aureus* (MRSA) strains without the development of antimicrobial resistance. Its cationic charge allows interaction with a negatively charged microbial cell wall that inhibits cell functions.^33^ OCT is primarily used in solutions or carbomer-based gels, which must be frequently reapplied, so there is room for developing a more advanced system for the delivery of the OCT. Current hydrogel formulations with an OCT (e.g., Octenisept) do not possess other requirements, such as adequate mechanical stability, an OCT-sustainable release, and biodegradability.

The present study provides insight into the synergic effect of OCT antiseptics, antibacterial natural polysaccharides, and hydrophilic synthetic polymers combined with advanced fabrication techniques, resulting in a sandwich-like transparent film. The integration of OCT into the hydrogel matrix exhibited sustained release and antibacterial action and addresses the growing challenge of antibiotic resistance by offering a broad-spectrum antimicrobial strategy.^34^ This novel hydrogel film formulation enhances its functionality, making it suitable for applications beyond wound care, such as in antibacterial coatings and infection-resistant materials.

## 2 Experimental Section

### 2.1 Materials

Gum Karaya (GK, Merck, Germany), sodium hydroxide (Lar-Nech, Czech Republic), hydrochloric acid (Lar-Nech, Czech Republic), ethanol 96% (Lar-Nech, Czech Republic), poly­(vinyl alcohol) (*M*_w_ = 130,000 g·mol^–1^, >99% hydrolyzed) (Merck), poly­(ethylene glycol) 400 (Merck, Czech Republic), glycerol anhydrous (Penta s.r.o., Czech Republic), Milli-Q ultrapure water Type I according to ISO 3696, normal saline (0.9% NaCl) (Braun, Germany), octenidine dihydrochloride, 98% (Alfa Aesar, China), Mueller Hinton Broth (Merck, U.K.), Dulbecco’s modified Eagle medium (DMEM), fetal bovine serum (FBS), penicillin/streptomycin, trypsin/ethylenediaminetetraacetic acid (EDTA), XTT ((2,3-bis­(2-methoxy-4-nitro-5-sulfophenyl)-2*H*-tetrazolium-5-carboxanilide)), and phosphate-buffered saline (PBS, Sigma-Aldrich, Germany) were used.

### 2.2 Methods

#### 2.2.1 Modification and Purification of Natural Gum Karaya

Prior to use, GK powder was deacetylated to increase its solubility in water following the deacetylation method described by Postulkova et al.^14^ The original GK powder was mixed with ultrapure water (obtaining a 2 wt % solution), placed on a magnetic stirrer at 300 rpm, and homogenized for 24 h at room temperature. Deacetylation of the solubilized original GK powder was provided by the addition of 1 mol·L^–1^ of sodium hydroxide (NaOH) in a ratio of three parts of the GK dispersion and one part of NaOH and stirred for 10 min at room temperature. The addition of NaOH caused an increase in the pH value to 12; to adjust the pH to neutral (7), a diluted solution of 0.3 mol·L^–1^ hydrochloric acid was used. The increase in solubility was carried out by deacetylation of the original GK, where hydroxyl groups replaced the acetyl groups. The modified GK was centrifuged at 25 °C and 3234 rcf to remove impurities. The remaining impurities were eliminated by filtration through polypropylene filters for better purification. Finally, the GK was then precipitated in ethanol in a 2:1 ratio and freeze-dried (freeze-dryer Epsilon 2-10D LSCplus, Martin Christ, Germany). The powder was frozen at −30 °C and then freeze-dried at −35 °C at a pressure of 1 mbar for 15 h. Then, secondary drying occurred at 25 °C under a pressure of 0.01 mbar until a decreasing Δ*p* (the pressure change was up to 10%). The dry sample of GK was then crushed into a powder and stored in a desiccator at room temperature.

#### 2.2.2 Hydrogel Films Fabrication by the Freeze-Drying Method

The modified GK powder was dissolved in ultrapure water, obtaining 2 wt % solutions after solubilization for 3 h under reflux at 90 °C to prevent evaporation of the vapor. A 4 wt % PVA solution was dissolved in ultrapure water under reflux for 2 h at 100 °C. Hydrogel solutions were prepared using different PVA and GK ratios (Table 1). The prepared solutions were homogenized with magnetic stirring at 300 rpm at room temperature overnight with the addition of PEG and glycerol. The ratios of solutions used for hydrogel films are summarized in Table 1. Based on glycerol concentrations, two distinct sample series were named: Series 1 (25 wt %) and Series 2 (75 wt %). The final polymer mixture was centrifuged at 3234 rcf to remove excess bubbles and impurities in the solution and then poured into Petri dishes and dried by the freeze-drying method, the same as used for GK powder. The final dry hydrogel films were stored in a desiccator at room temperature.

**Table 1:** Summary of the Sample Composition^a^

	polymers wt % in hydrogel solution		hydrogel composition in the dry state			
sample name	GK	PVA	(GK/PVA) ratio		glycerol amount (wt %)	OCT concentration (μg·cm^–2^)
1GPgL	1.25	1.25	1	1	25	
1GPgH	1.06	1.06	1	1	75	
2GPgL	0.93	1.87	1	2	25	
2GPgH	0.78	1.55	1	2	75	
3GPgL	1.43	0.95	1.5	1	25	
3GPgH	1.26	0.84	1.5	1	75	
1GPgH-oL	1.06	1.06	1	1	75	0.05
1GPgH-oH	1.06	1.06	1	1	75	0.1
2GPgH-oL	0.78	1.55	1	2	75	0.05
2GPgH-oH	0.78	1.55	1	2	75	0.1

a The first number (1, 2, or 3) in marking samples represents the different ratio between GK and PVA; G means GK, P states for PVA, gL means 25 wt % glycerol, gH is for 75 wt % glycerol, oL means an OCT of 0.05 μg·cm^–2^, and oH means an OCT of 0.1 μg·cm^–2^ concentration.

#### 2.2.3 Hydrogel Film Coating by Antimicrobial OCT

OCT stock solutions were prepared by dissolving it in ethanol (96%) at given concentrations (375 and 750 μg·mL^–1^) and stirred until homogenized. Two materials (1GPgH and 2GPgH) were used for the coating with OCT concentrations of 0.05 and 0.1 μg·cm^–2^, as shown in Table 1, according to the preferred concentrations for treating chronic wounds and multidrug-resistant organisms.^35^ The hydrogel films were coated by applying a defined amount of an OCT solution onto the hydrogel surface. Subsequently, the coated hydrogel films were dried at room temperature, followed by evaporation of the remaining ethanol at 60 °C, preventing hydrogel film degradation. The coated hydrogel film was sealed in plastic plates and stored at room temperature.

#### 2.2.4 Optical Properties

The color values of the hydrogel films with different GK contents were determined using the CIELAB color system on a UV–vis Spectrophotometer V-730, JACSO. The CIELAB system expresses color and lightness as three parameters: L*, a*, and b*. L* characterizes lightness, describing the relative intensity of light with values from 0 in absolute black to 100 in absolute white and can be used to determine the material transparency. The other two parameters describe a color coordinate: a* from green (−) to red (+) and b* from blue (−) to yellow (+). The difference between the samples in the dry and hydrated states can be objectively evaluated using color difference *E* in the following equation

1 $$E=\sqrt{L^{2}+a^{2}+b^{2}}$$

The *L*, *a*, and *b* represent the color parameter values of dry or hydrated hydrogel film samples at the water equilibrium to compare the lightness and the color difference *E* in the wavelength region of 800–380 nm in a quartz cuvette with the background of the empty cuvette.

The opacity of the hydrogel film was determined using a UV–vis spectrometer, measuring the light absorbance of dry/hydrated hydrogel films at 600 nm (*A*_600_) against air in an empty cuvette. Hydrogel film opacity was obtained using eq 2 ^36^

2 $$\mathrm{opacity}=\frac{A_{600}}{d}$$

Here, *d* states the thickness of the sample in the dry and hydrated states. Graphs and statistics were created in OriginPro2020b. Each measurement was repeated five times to obtain a reliable standard deviation.

#### 2.2.5 Swelling Behavior

The gravimetric method was used to determine the swelling behavior of freeze-dried and coated hydrogel films. Testing was carried out in normal saline (0.9% NaCl) at a temperature of 37 °C. All samples were cut into circular shapes with approximately the same weight. The samples were placed in a Petri dish and weighed in a dry state before testing. The measurement then occurred at the following intervals: 1, 5, 10, 20, 40, 60, 90, and 120 min. All samples were placed in the incubator to keep the temperature at 37 °C. Before each weighing, the surface water on the sample was removed by filtration paper, and after that, the sample was weighed on analytical balances. The swelling behavior was determined by the swelling ratio (SR) using eq 3

3 $$swelling\;ratio\;\left[\text{−}\right]=\frac{w_{s}-w_{d}}{w_{d}}$$

The dry sample and that of *w*_d_ represent the weight of *w*_s_, which represents the swollen state of the hydrogel film sample in the equation. Each measurement was repeated five times to obtain a reliable standard deviation.

#### 2.2.6 Hydrogel Film Weight Loss

Hydrogel film degradation was evaluated with samples immersed in Milli-Q type I ultrapure water and incubated at 37 °C. First, the material was weighed to obtain *w*_max_ (weight of the swelled material in time intervals correlated to the maximum weight of the swelled sample, specific for each sample). Subsequently, the weight was measured in time intervals of 1, 3, 7, 14, and 28 days, obtaining *w*_s_. Hydrogel film degradation was evaluated using eq 4

4 $$w\mathrm{eight loss}\;\left[\%\right]=100-\frac{w_{s}\times 100}{w_{\max}}$$

Each measurement was repeated four times.

#### 2.2.7 Attenuated Total Reflectance-Fourier-Transform Infrared Spectroscopy (ATR-FTIR) Analysis

The presence of the functional groups on the surface and bulk of the samples was confirmed by Fourier-transform infrared spectroscopy with attenuated total reflectance (ATR-FTIR) with an ATR-FTIR Vertex 70/70v spectroscope (Bruker). Samples were measured in the form of hydrogel films, raw material (GK, PVA) in powder form, and coated hydrogel films. The infrared spectrum was scanned between 3700 and 700 cm^–1^ with the number of scans equal to 32 and a resolution of 2 cm^–1^.

#### 2.2.8 Thermogravimetric Analysis (TGA)

The hydrogel film and OCT-coated films’ thermal stability was characterized by thermogravimetric analysis performed using Discovery TGA, TA Instruments, Nicolet iS10 (Thermo Fisher Scientific). The heating rate was set at 10 °C·min^–1^ in a nitrogen atmosphere on platinum plates ranging from 40 to 700 °C.

#### 2.2.9 Tensile Strength Test

The tensile strength and elongation of the swollen hydrogel films were determined by using the RSA-G2 TA rheometer. The dried hydrogel films were cut into strips (3 × 0.5 cm^2^), placed between the grips, and swelled in water at 37 °C until they reached the maximum swelling ratio. Then, the measurement was performed at a constant linear rate set at 0.2 mm·s^–1^ until the sample reached the breaking point.

#### 2.2.10 Hydrogel Film Morphology

The surface microstructure of noncoated hydrogel film samples was studied using advanced environmental scanning electron microscopy (A-ESEM).^37,38^ In-house modified A-ESEM QUANTA 650 FEG (Thermo Fisher Scientific) was used under operating conditions: a beam accelerating voltage of 5 kV, beam current of 53 pA, working distance of 8.5 mm, and 200 Pa of water vapor pressure, using an Ionization Secondary Electron Detector with an electrostatic Separator (ISEDS).^39,40^ The thickness measurement of an antiseptic layer was carried out on freeze-fractured samples. The hydrogel film pieces were snap-frozen in liquid nitrogen, fractured at −196 °C, and then fixed on the tilted stub. For detailed observation of hydrogel film pores, samples were in situ freeze-dried using the A-ESEM. Small hydrogel film samples (2 × 2 mm^2^) were placed on a cooled specimen holder (Peltier stage) and cooled to −20 °C (cooling rate of 20 °C·min^–1^). When a temperature of 0 °C was reached, the pumping process of the A-ESEM specimen chamber started, according to the low-temperature method for ESEM (LTM) described by Neděla et al.^41^ and used for the study of various samples.^42,43^ The samples were observed under the following operating conditions: specimen holder temperature of −20 °C and water vapor pressure of 150 Pa. The pore size of the prepared hydrogel films was characterized by A-ESEM visualization using the image analysis program SEM Image Pore Extractor (SEMIPE),^44^ an automatic tool for pore-dimensional extraction from SEM images of freeze-dried hydrogel films.

#### 2.2.11 Energy-Dispersive X-ray Spectroscopy (EDS) Elemental Analysis

Semiquantitative energy-dispersive X-ray microanalysis (EDS) was performed to detect the presence of an antiseptic OCT. Analysis was carried out on freeze-dried samples (freeze-dryer Epsilon 2-10D LSCplus, Martin Christ, Germany). Chemical treatment-free and conductive coating-free samples were analyzed using a Bruker Quantax 400 XFlash 6/60 EDS silicon drift detector under a beam energy of 5 keV, beam current of 100 pA, working distance of 10 mm, and water vapor pressure of 200 Pa. The EDS spectra were obtained from five regions of each sample.

#### 2.2.12 OCT Quantification and Release

OCT was quantified using a UV–vis V-730 Spectrophotometer, JACSO at 280 nm. The stock solution contained 1% OCT; the final concentrations for the calibration curve were in the range of 0.01–1.5 μg·mL^–1^ of OCT in ethanol.

The release of the OCT coated at various concentrations on the hydrogel film surface of two sample types (1GPgH and 2GPgH) was studied at 37 °C in a shaking incubator immersed in 1 mL of ultrapure water. At given time intervals (30 min, 1, 2, 4, 6, 10 h, and 1, 3, 5, 7, 9, 12, and 14 days), all of the solutions with released OCT were removed for quantification analysis using UV–vis spectroscopy from the OCT calibration curve described above. The removed water was replaced with the same amount of ultrapure water preheated to 37 °C. The graphs were created in OriginPro2020b. Each measurement was repeated three times.

Release data were applied to various mathematical drug release models to investigate the drug release kinetics of the OCT. Two mathematical models were used in this study to obtain the *R* ^2^ value.

(a) Higuchi model, which can be represented using eq 5
5 $$\frac{M_{t}}{M_{\infty }}=K_{h}t^{1/2}$$
  where *M*_*t*_/*M*_*∞*_ represents the fraction of released drug in each time interval (*t*), *M*_*t*_ represents the amount of the drug at time *t*, *M*_*∞*_ is the amount of drug released after time *∞*, and *K*_h_ is represented as the Higuchi release kinetic constant.
(b) Korsmeyer–Peppas (KP) model, which can be interpreted as eq 6
6 $$\frac{M_{t}}{M_{\infty }}=K_{\mathrm{kp}}t^{n}$$
  where *M*_*t*_/*M*_*∞*_ represents the fraction of released drug in each time interval (*t*), *M*_*t*_ represents the amount of the drug in time, *M*_*∞*_ is the amount of drug released after time *∞*, *n* is a drug release exponent, and *K*_kp_ is the Korsmeyer release rate constant.

#### 2.2.13 Cell Culture and Cytotoxicity Assay

NIH-3T3 fibroblast cells (Sigma-Aldrich) were cultured in DMEM medium supplemented with 10% FBS and 1% penicillin/streptomycin at 37 °C and 5% CO_2_. Cells were harvested by trypsinization in a 0.25% trypsin/EDTA solution in PBS at 80% confluence. The extract test was used to evaluate the cytotoxic effect of any leachable components or byproducts of the hydrogel film. Each film was incubated with a complete DMEM culture medium at room temperature at 0.033 g·mL^–1^ for 24 h. NIH-3T3 cells were seeded at a density of 10^4^ cells per well in a 96-well plate prior to starting the assay. After 24 h of cell growth, the cell culture medium was removed and replaced with the extract medium. The cells were then incubated at 37 °C and 5% CO_2_ for 24 h. The XTT assay was used according to manufacturer’s protocols. The extract medium was removed from the well plate, and the cells were gently washed using a PBS buffer solution. 100 μL of fresh DMEM medium and 50 μL of XTT (XTT, 1 mg·mL^–1^ in PBS, pH 7.4) labeling mixture was then added per well. The absorbance was measured after 4 h of incubation at 37 °C with a plate reader at 450 nm.

#### 2.2.14 Antibacterial Activity

Bacterial strains *S. aureus* CCM 3953 (=ATCC 25923) and *Escherichia coli* CCM 3954 (=ATCC 25922) obtained from the Czech Collection of Microorganisms were utilized. Both bacterial strains serve as standard international reference strains for antimicrobial susceptibility testing. Susceptibility testing was performed according to the standard broth microdilution methodology outlined by EUCAST with some modifications.^45^ The tested materials were placed in triplicate in a 24-well microplate (JET BIOFIL 24 Well, Guangzhou, China). Both bacterial strains were grown overnight in 10 mL of Mueller Hinton Broth (MHB; Oxoid, Merck, U.K.) at 37 °C. The overnight cultures were then centrifuged and resuspended in fresh MHB twice. The bacterial cultures were then diluted with fresh MHB to obtain a bacterial suspension with a final concentration of 5 × 10^5^ CFU·mL^–1^. 1 mL portion of the suspension was added to the tested materials and sealed with sterile ThermalSeal films (Sigma-Aldrich, St. Louis, MO). The microplates were incubated at 37 °C with shaking in a Tecan Infinite M200 PRO microplate reader (Tecan Trading AG, Trasadingen, Switzerland), and the optical density (*A* = 600 nm) of the bacterial suspension was measured every 5 min for 24 h to obtain bacterial growth curves.

#### 2.2.15 Statistical Analysis

Statistical analysis of the data was performed using the OriginPro2020b and Statistica 64 programs. For each parameter, the data were expressed as mean values ± standard error of the mean of at least three samples (porosity, swelling, degradation, mechanical, antibacterial, and cytotoxic tests). Analysis of variance (ANOVA) and partial correlation analysis, Tukey’s test, and the *t* test for dependent samples were performed using a 95% significance level to evaluate the significance level of different sample types for various analyses, including porosity, mechanical tests, and antibacterial tests; *P < 0.05, **P < 0.01, and ***P < 0.001 were considered to indicate statistically significant results.

## 3 Results and Discussion

### 3.1 Optical Properties of Freeze-Dried Hydrogel Films

Color evaluation and opacity tests were performed on dry and hydrated film samples to determine whether the hydrogel films could provide direct, transparent monitoring. Table 2 shows that L*, a*, and b* represent the hydrogel films’ lightness, redness, and yellowness, depending on the hydrogel film composition or the state (dry/wet).

**Table 2:** CIELAB Color Space Determines the Color of the Hydrogel Films (L*, Lightness; a*, Red; and b*, Yellow Opponent Values), Total Color Difference E*, and Materials’ Opacity

sample	hydrogel film state	L*	a*	b*	E*	opacity
1GPgL	dry	0.5	0.1	0.8	0.9	4.3
	hydrated	58.6	1.2	1.8	58.6	0.7
2GPgL	dry	0.8	0.0	0.9	1.2	2.5
	hydrated	22.4	1.3	4.6	22.9	2.7
3GPgL	dry	1.4	0.1	1.1	1.8	4.8
	hydrated	43.7	3.5	19.1	47.8	2.1
1GPgH	dry	76.6	1.0	10.9	77.4	1.0
	hydrated	40.5	2.7	11.0	42.1	2.0
2GPgH	dry	67.2	1.6	13.2	69.0	1.6
	hydrated	42.8	2.1	7.0	43.4	1.2
3GPgH	dry	71.4	0.2	8.2	71.9	1.5
	hydrated	42.2	3.7	19.7	46.7	1.5

The results show that the hydrogel films’ redness (a*) and yellowness (b*) are not significantly different in most films within the same series and state. However, the CIELAB values differ within the different dry and hydrated states and in series with *P*-values > 0.05. The composition strongly influences the lightness of the film, and the samples differ significantly (*P* > 0.05). The total color difference increases significantly in all samples after water absorption, causing a considerable change in the visual aspect, as shown in Figure 1. The Series 1 sample (25 wt % glycerol) has a whitish color in the dry state due to the presence of a highly porous network that becomes partially transparent in the hydrated state, followed by a slight increase in b*, indicating a yellowish color. The increase in transparency in hydrated samples correlates with a decrease in opacity and a significant increase in hydrogel film lightness (L*), accounting for ∼117 times higher L* when hydrated compared to the dry state, and E* is ∼65 times higher. Sample 2GPgL has comparable E* and L*, accounting for ∼18 times higher hydrated state values than in the dry state, which is influenced by a higher PVA concentration that creates a dense and porous polymer network between PVA, GK, and PEG chains with glycerol. The hydrated state of hydrogel films is highly impacted by the swelling behavior, which leads to the expansion of the hydrogel film network and pore enlargement and completely changes the visual aspect of the hydrogel film with a low glycerol content (25%). Porous freeze-dried hydrogel films in a hydrated state are generally transparent and rigid materials, as previously described by Sedlář et al.^46^ In contrast, samples of series with gH (75% glycerol) have high L* (<67) and E* (<70) values in the dry state, indicating hydrogel’s transparency, which is visualized in Figure 1 and can be attributed to the presence of optically transparent micropores and smooth hydrogel surfaces in the dry state. A high E* value in Series 2 is highly influenced by the high value of L* and a higher yellowish b* (<8) value. Hydrated samples slightly decrease L*, indicating a decrease in transparency, which cannot be compared with Series 1. However, the samples are transparent enough to visualize the surface under the hydrogel (Figure 1), and a decrease in E* is comparable with L*, accounting for 1.5–1.8 times lower values when hydrated. A slight increase in opacity when samples are hydrated can be attributed to the enlargement of the porous network beneath the hydrogel surface with entrapped water within the matrix and glycerol.

**Figure 1:**
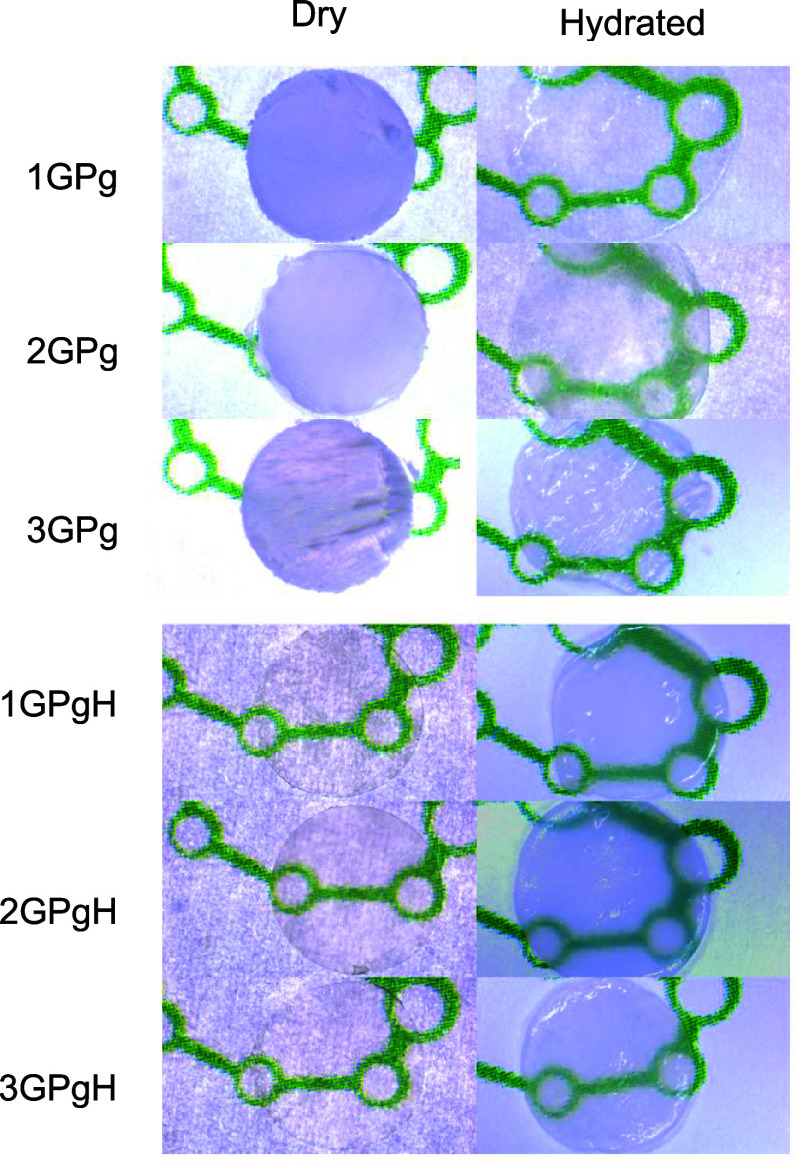
Visual aspect of hydrogel films 1GPgL, 2GPgL, 3GPgL, 1GPgH, 2GPgH, and 3GPgH in the dry and hydrated state.

### 3.2 Swelling Behavior of Freeze-Dried Films

The ability to swell and retain water is a crucial property of hydrogel films in creating a moist environment. Swelling behavior is summarized in Figure 2A, while hydrogels with coated OCT are shown in Figure 2B. All hydrogel films tended to swell in normal saline and retained it within the structure, providing additional stability during the experiment (120 min). A higher swelling rate was observed for samples 1GPgL and 2GPgL containing 25 wt % glycerol. These samples had a suitable ratio between PVA and glycerol, creating a stable polymer network through hydrogen bonding interactions, crystalline domains of PVA, and polymer chain entanglement between GK and PVA. From Figure 2A, sample 2GPgL exhibited a higher swelling ratio (SR), reaching 4.5 after 20 min, with overall stability due to a higher PVA concentration (GK/PVA ratio of 1:2), creating a stable network able to absorb and preserve liquid. In comparison, sample 1GPgL swells more rapidly, reaching its peak after 10 min with SR = 3.8, with a sustainable decrease in SR over time. This indicates that a ratio of 1:1 between polymers and 25% glycerol results in a less robust yet swellable polymer network, with the tendency to partially dissolve. This behavior is likely due to the higher GK content, which is not strongly integrated via hydrogen bonding with other components. Instead, the polymer chains of GK and PVA are primarily held together through physical entanglement, leading to a reduced structural stability. According to the results, the highest water absorption was observed in sample 3GPgL, accounting for SR = 5.5, showing an increasing trend due to a high concentration of swellable GK, which preferentially interacted with the liquid environment and promoted hydrogel swelling. Simultaneously, hydrogen bonding interaction between PVA and glycerol provided a sufficiently strong cross-linked network that prevented hydrogel dissolution, effectively entrapping GK.^14^ The least stable and swellable hydrogel was sample 3GPgH, with a higher concentration of glycerol (75%) and swellable GK (GK/PVA ratio of 1.5:1). The polymer network lacked sufficient stability due to the low PVA content, which hindered formation of hydrogen bonds between PVA and glycerol, limited entanglement of PVA and GK, and reduced creation of PVA crystalline domains. These factors led to rapid initial swelling and subsequent dissolution in the hydrated environment, as GK and glycerol interacted more with the surrounding liquid than created a hydrogel network.

**Figure 2:**
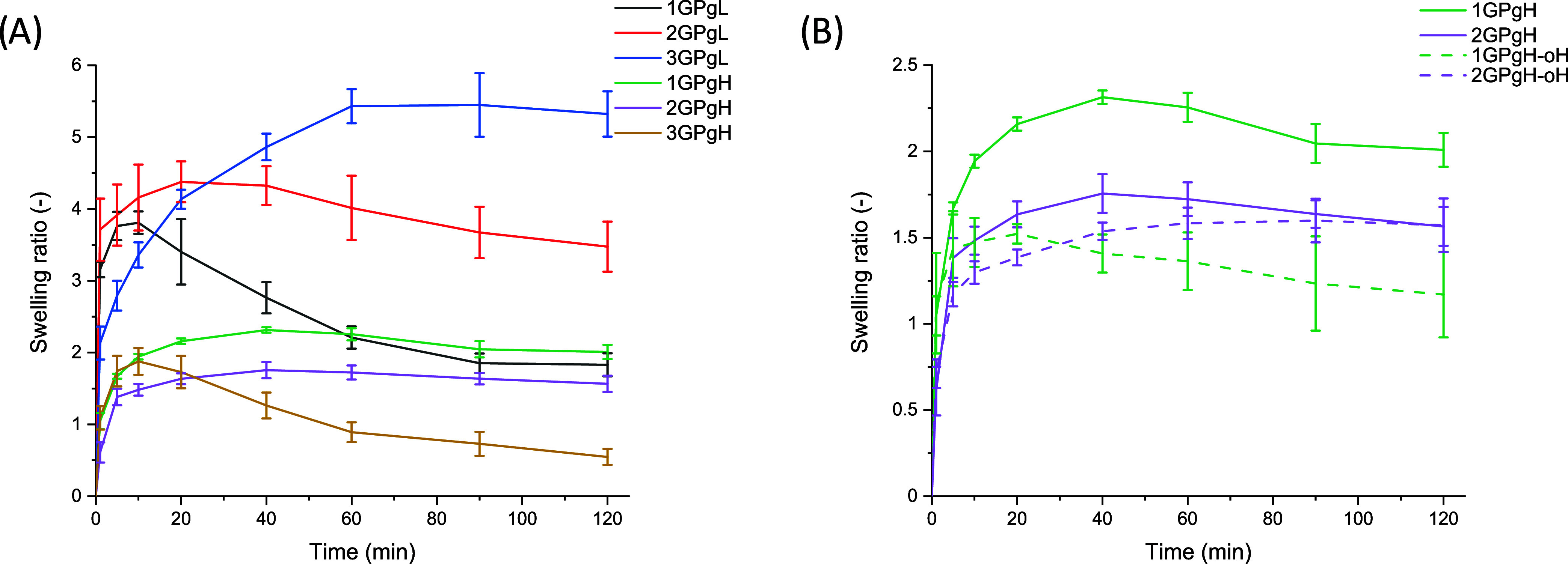
Swelling ratio of prepared hydrogel dressings depending on time (A) and of OCT-coated hydrogels (B).

In comparison, samples with 25% glycerol, 1GPgH and 2GPgH (75% glycerol) exhibited significantly lower SR, likely due to the higher glycerol content, which can contribute to swelling-resistance properties and increased hydrogel flexibility while limiting absorption.^47^ Both samples had a visibly nonporous surface, likely composed of a glycerol–PVA layer, which enhanced glycerol swelling-resistance properties. This resulted in a gradual mass increase during SR measurement and helped retain liquid within the polymer network while reducing hydrogel degradation. Despite having a high glycerol content of 75%, the presence of crystalline domains in PVA, hydrogen bonding between PVA and glycerol, and polymer chain entanglement was sufficient to maintain a stable polymer network. Although a dense glycerol layer was present on the hydrogel surface, GK swelling is evident. Hydrogel 1GPgH showed a slightly higher swelling, accounting for SR = 2.4 after 40 min, compared to SR = 2 in hydrogel 2GPgH at the same time. The effect of PEG was not prominently observed in any of the samples as its concentration remained constant across formulations. PEG, similar to glycerol, acts as a plasticizer, enhancing hydrogel flexibility and reducing brittleness; thus, its impact is not discussed further. However, due to its uniform presence, its influence on swelling behavior was considered negligible and is therefore not discussed further. The addition of the OCT did not significantly affect the swelling behavior; although the coating process was being performed in ethanol, which precipitates GK, it partially altered the hydrogel surface. Sample 2GPgH-oH had a slightly lower swelling capacity than 2GPgH, while sample 1GPgH-oH in Figure 2B exhibited a much lower swelling than 1GPgH. The altered surface caused a slightly reduced swelling capacity and decreased sample stability, which may have impacted OCT release.

### 3.3 Hydrogel Film Hydrolytic Stability

The hydrolytic stability of the hydrogel films was evaluated by measuring the mass loss over time in ultrapure water in an incubator at 37 °C for 28 days. Figure 3 illustrates the weight loss trends, indicating that the hydrogel stability strongly depends on the formation of a stable network between the components. Sample 3GPgH was excluded from the experiment due to its instability and rapid disintegration. All tested samples demonstrated stability throughout the measurement period, although their overall stability varied depending on the composition. The hydrogels can be categorized into two distinct groups: porous samples with lower glycerol content and transparent, smooth samples with higher glycerol content. Hydrogels 1GPgH and 2GPgH remained stable during the whole measurement, with a weight loss accounting for 50%. The smooth, nonporous surface of these samples containing 75% glycerol likely contributes to their stability by entrapping and preserving fluid within the bulk network. Surface glycerol is known to promote swelling resistance, which may have further enhanced their hydrolytic stability. The minor difference in hydrolytic stability of hydrogels with 75% glycerol indicates GK/PVA ratio is not the primary determinant of stability in high-glycerol systems. A high concentration of glycerol facilitates hydrogen bonding with PVA, which simultaneously limits the creation of crystalline domains, thereby reducing the rigidity of the hydrogel. PVA and glycerol hydrogen bonding at the same time reinforce polymer chain entanglement between PVA and GK, creating a hydrogel that is hydrolytically stable over time. This trend appears only specifically in hydrogels with 75% glycerol, which form a robust network. In contrast, hydrogels with 25% glycerol have a different hydrolytic stability behavior trend. Among them, hydrogel 2GPgL with a higher PVA concentration behaves similarly to the high-glycerol samples. The elevated PVA content (ratio of GK and PVA is 1:2) promotes the formation of a crystalline domain within PVA chains, resulting in a rigid, sponge-like, and highly porous hydrogel. At the same time, hydrogen bonding occurs primarily between PVA chains and glycerol, stabilizing the hydrogel network independently of GK–PVA entanglement. This leads to a highly stable hydrogel with a lower tendency to swell and dissolve.^6^ Hydrogels 1GPgL and 3GPgL, both containing 25% glycerol, exhibited a higher weight loss, likely due to a higher GK content. This composition supports liquid sorption and subsequent dissolution, as previously described by Drápalová et al.^48^ After rapid swelling, the matrix weakens and GK is washed from the hydrogel network, resulting in a decrease in weight. Nevertheless, the presence of PVA crystalline domains and hydrogen bonding between PVA and glycerol prevented complete degradation, allowing these samples to remain partially stable throughout the experiment.

**Figure 3:**
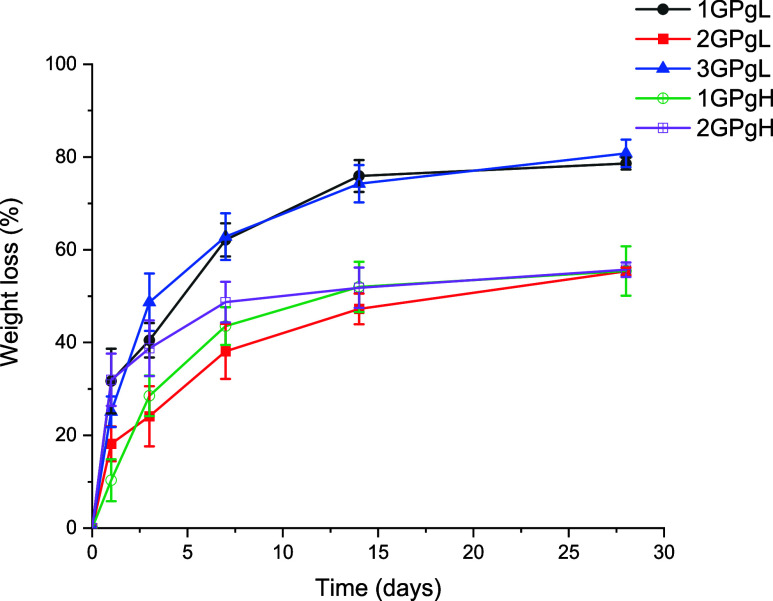
Hydrogel film weight loss for a 28-day experiment in type I ultrapure water at 37 °C placed in an incubator.

### 3.4 Chemical Compositions Determined by ATR-FTIR

ATR Fourier-transform infrared (ATR-FTIR) spectroscopy was used to characterize specific chemical groups present in the hydrogel films and to study the effect of composition to confirm the presence or absence of these groups in the chemicals and prepared hydrogel films. Spectra of raw gum Karaya and PVA were already published;^18^ the prepared freeze-dried hydrogel film spectra are shown in Figure 4A. Since hydrogel films are formed by freeze-drying, the hydroxyl groups present in PVA, glycerol, and GK are capable of bonding via hydrogen bonds and are expected to create specific interactions that influence the final spectra of the hydrogel. All samples show a wide absorption band at 3660–3080 cm^–1^, representing the hydroxyl stretching of GK, PVA, and glycerol with varying intensity, which is directly proportional to the number of present hydroxyl groups in the samples, as shown in Figure 4A. These samples tend to have a higher absorption band of the hydroxyl groups, indicating that not all of the hydroxyl groups are cross-linked via hydrogen bonding. On the contrary, hydrogel films 1GPgL, 2GPgL, and 3GPgL (25 wt % glycerol) have a lower intensity of −OH stretching (Figure 4A), corresponding to a lower presence of free hydroxyl groups and a higher formation of hydrogen bonding between components.^17,49^ The aliphatic C–H bonds of PVA are indicated by the band at 2940 cm^–1^. In particular, the vibrations at 1605 and 1418 cm^–1^ belong to the vibrations of the carboxylate group due to the carboxylation of the uronic acid residues of GK. The vibration at 1420 cm^–1^ represents the −OH alcohol groups of PVA. The broad absorption band at 1150–980 cm^–1^ indicates the presence of the characteristic C–O bond for the pyranose ring belonging to GK and the C–O bonds in PVA, PEG, and glycerol. Figure 4B compares the FTIR spectra of raw and OCT-coated hydrogel films. The spectrum of raw octenidine shows an absorption band at 2935 cm^–1^ that represents aliphatic C–H bonds. Another relevant peak is observed at 1654 cm^–1^, which represents the aromatic C=N groups. Vibrations with a band at 1190 cm^–1^ indicate the C–N stretching of the OCT. Aside from the aliphatic C–H bonds present in the OCT, samples with coated OCT have an almost identical spectrum to the original hydrogel film, the OCT peak at 1654 cm^–1^ is in the hydrogel film slightly shifted to the lower wavelengths at 1607 cm^–1^, proving the presence of the OCT. Low absorbance of the OCT absorbance in the sample might be caused by its low concentration and the fact that the OCT is present primarily in the dense hydrogel surface layer, and FTIR detects the bulk instead of the upper hydrogel layers. Moreover, the presentation of the OCT on the hydrogel surface was confirmed also by EDS analysis, as described later (see Table 3). Overall, the characteristic bands represented by all components were confirmed in the hydrogel matrix; however, various ratios between components clearly show, in normalized curves, that the corresponding signal to each component relies on its concentration in the dry hydrogel, although ATR-FTIR is not a primarily quantitative method.

**Figure 4:**
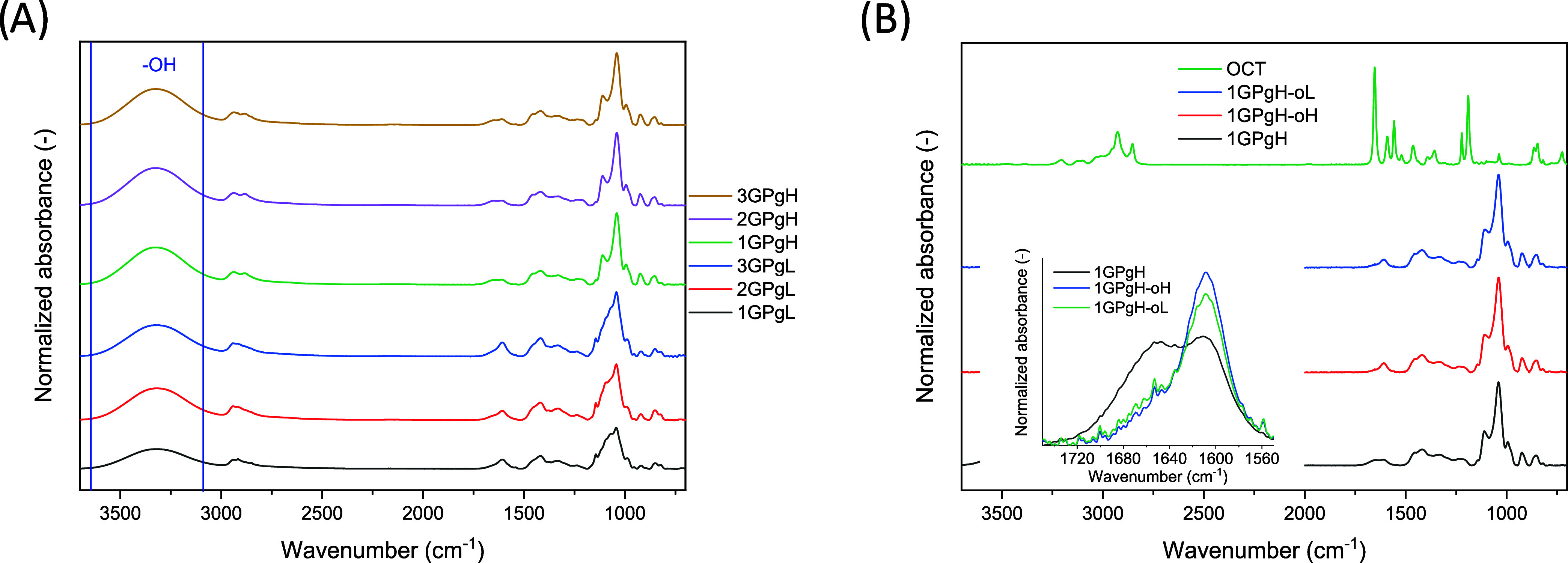
FTIR spectra of the prepared hydrogel dressing with different ratios between components (A), spectra of 1GPgH hydrogel, OCT, and coated hydrogels 1GPgH-oL and 1GPgH-oH (B).

**Table 3:** Analysis of the Content of Elements on the Hydrogel Surface Performed by EDS

sample	N (%)	Cl (%)
1GPgH	0.8	0.0
1GPgH-oL	1.0	0.1
1GPgH-oH	1.1	1.3
2GPgH	1.1	0.0
2GPgH-oL	0.8	0.1
2GPgH-oH	1.0	0.1

### 3.5 Thermal Stability of Hydrogel Films

The thermal stability of the hydrogel films was instrumentally measured by using thermogravimetric analysis (TGA) under a nitrogen atmosphere. Figure 5A shows the thermal degradation of the chemicals, which can be compared with the degradation steps of the prepared hydrogel films in Figure 5B,C and coated hydrogel films in Figure 5D. The first mass loss occurred at temperatures below 200 °C, attributed to bound water. For Series 1 (25% glycerol) (Figure 5B), the hydrogels exhibit two-step degradation with similar curve shapes for samples 1GPgL, 2GPgL, and 3GPgL in the 90–350 °C range. The first peak can contribute to the decomposition of glycerol and water residues; the second peak represents the decomposition of PVA and GK with solid residues at 700 °C, with 26.38, 23.07, and 29.77 wt % stable GK residues. The mass loss in Series 2 (75 wt % glycerol) (Figure 5C) degrades mainly in the range of 110–205 °C, indicating the decomposition step of glycerol due to its high concentration and bound water. The second stage of decomposition is for PVA and GK in the range of 270–370 °C, with the remaining solid residues of GK and PVA being 10.17 wt % (1GPgH), 11.74 wt % (2GPgH), and 13.51 wt % (3GPgH). The cross-linked network between PVA, GK, and glycerol did not significantly impact the thermal stability of the hydrogel film; only the mass concentration and the glycerol layer on the surface could affect the thermal stability. The percentage of mass corresponds to the weight ratio of each component in the hydrogel. Overall, the thermal degradation of all tested samples is directly proportional to the hydrogel composition, so physical cross-linking via hydrogen bonding, PVA crystalline domains, and polymer chain entanglement do not significantly influence this kind of degradation and overall thermal stability. Both coated hydrogel films in Figure 5D have the same initial weight loss as the original hydrogel films due to the presented bound water. The second and third decomposition steps have parallel curves with uncoated films, showing that the OCT has no significant impact on the hydrogel thermal stability due to its low concentration. The only difference between noncoated and coated films is the larger mass of residues after the measurement, which the presence of the OCT might cause. However, there were no additional TGA measurements of the OCT because of minimal differences. From the results, we can conclude that the OCT coating had no impact on hydrogel composition and cross-linking and did not affect the thermal decomposition of the hydrogel film.

**Figure 5:**
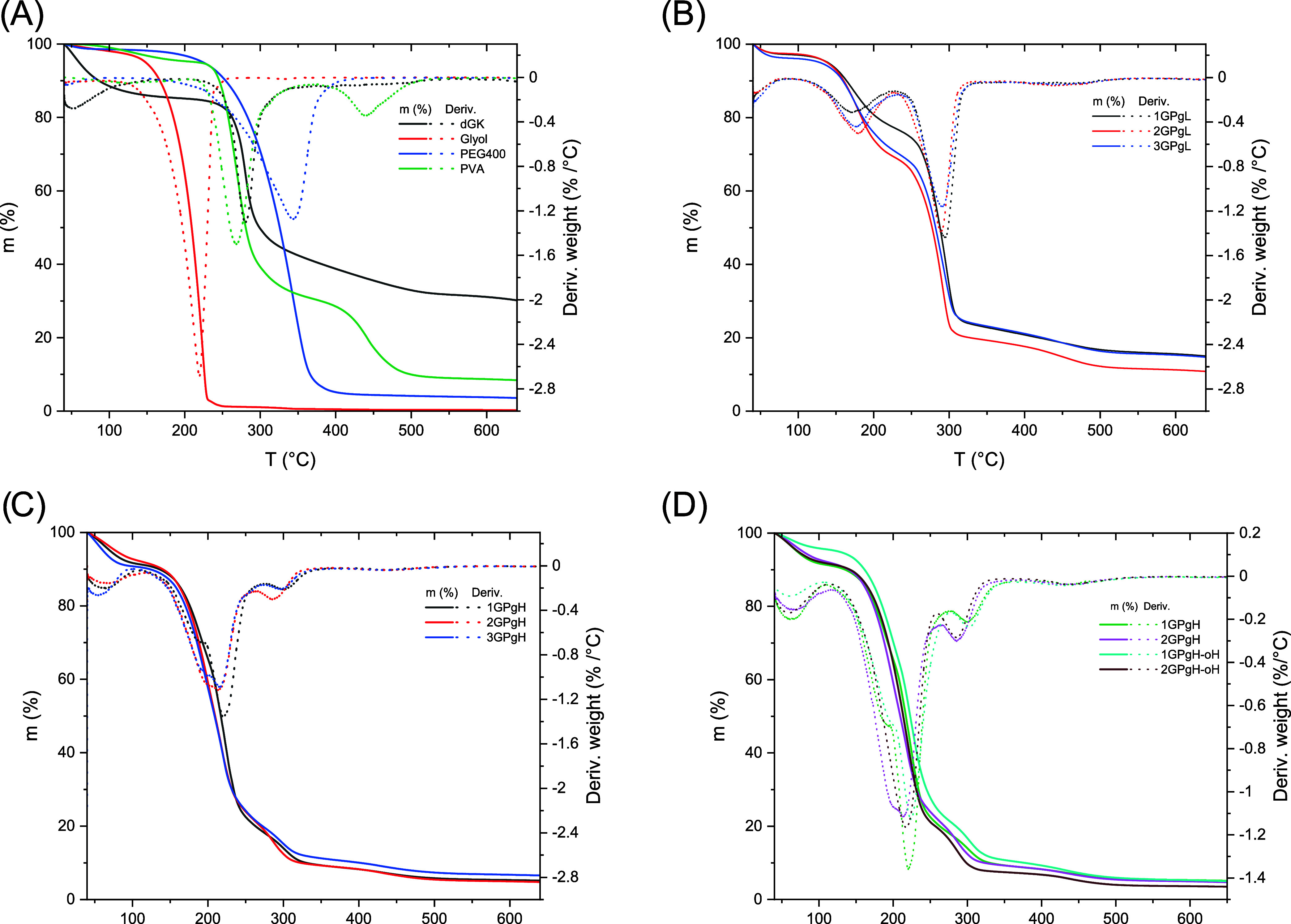
TGA curves and derived weight curves of raw materials (A), hydrogel films with different compositions (B), GK hydrogels (C), and OCT-coated hydrogel films (D). *m* in the graph represents mass loss.

### 3.6 Mechanical Properties of Hydrogel Films

The tensile strength behavior of the hydrogel films was evaluated in the hydrated state to determine the stretchability and endurance of the samples while handling. The test was carried out at 37 °C in ultrapure water; the hydrogel was mounted in two grips and hydrated until equilibrium; sample 3GPgL was excluded due to its brittle state in the dry state. The average tensile curves of the hydrated films are presented in Figure 6, showing similar curve shapes across samples, despite differences in breaking points.

**Figure 6:**
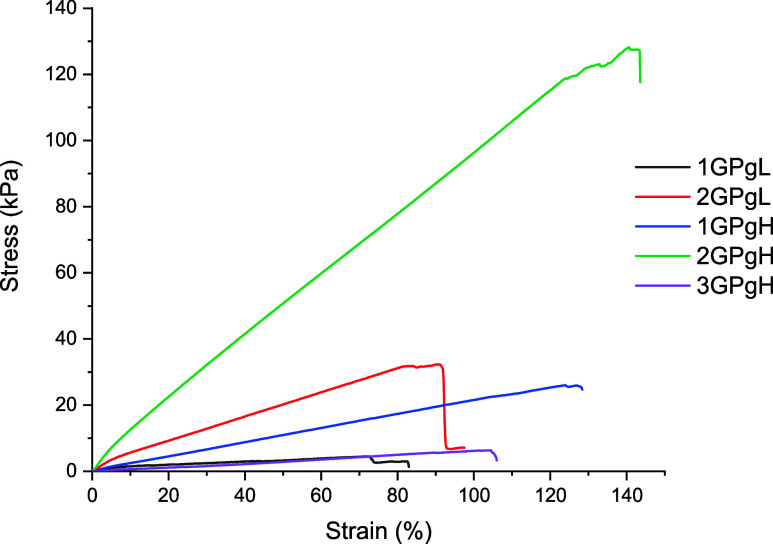
Average tensile curves for various sample compositions in water at 37 °C after equilibrium swelling degree of the hydrogels.

As seen in Figure 6, sample 2GPgH (75 wt % glycerol) exhibited the highest tensile strength and elasticity, with a breakpoint of 128.7 ± 11.37 kPa. This performance is attributed to its optimized composition, where a high PVA content and glycerol concentration form a dense, elastic polymer network through hydrogen bonding and chain entanglement. Formation of crystalline domains is neglected due to the high glycerol content, reducing the sample rigidity. In contrast, the remaining samples showed significantly lower tensile strength, averaging 31.7 ± 2.1 kPa, approximately 4 times lower than 2GPgH. The influence of composition is evident: sample 2GPgL, with reduced glycerol but higher PVA content, showed the second-highest toughness. PVA contributes to a rigid yet strong network, and in this case, the plasticizing effect of glycerol is minimized. Sample 1GPgH, with a 1:1 GK/PVA ratio and high glycerol content, demonstrated moderate strength but high elasticity, with an elongation at break of 139.1 ± 7.0%. This suggests that while glycerol enhances flexibility, the reduced PVA content compromises mechanical strength. The weakest mechanical performance was observed in sample 1GPgL, which combined low glycerol content with a 1:1 GK/PVA ratio. This composition resulted in a brittle hydrogel, consistent with the limited mechanical contribution of GK and the rigidity of PVA in the absence of a sufficient plasticizer. Similarly, sample 3GPgH, with high concentrations of both GK and glycerol, formed an elastic but mechanically unstable hydrogel, likely due to insufficient PVA to support network integrity.

Figure 7A,B summarizes the mechanical performance of the hydrogels, highlighting the influence of composition on the tensile strength and elongation. As previously discussed, sample 2GPgH exhibited the highest stiffness and elasticity due to its optimized PVA and glycerol content. In contrast, samples with higher GK content or lower PVA showed reduced mechanical strength. Glycerol consistently enhanced elongation, while GK contributed to structural weakening. These results reinforce the importance of balancing PVA, glycerol, and GK to tailor hydrogel mechanical properties for specific applications.

**Figure 7:**
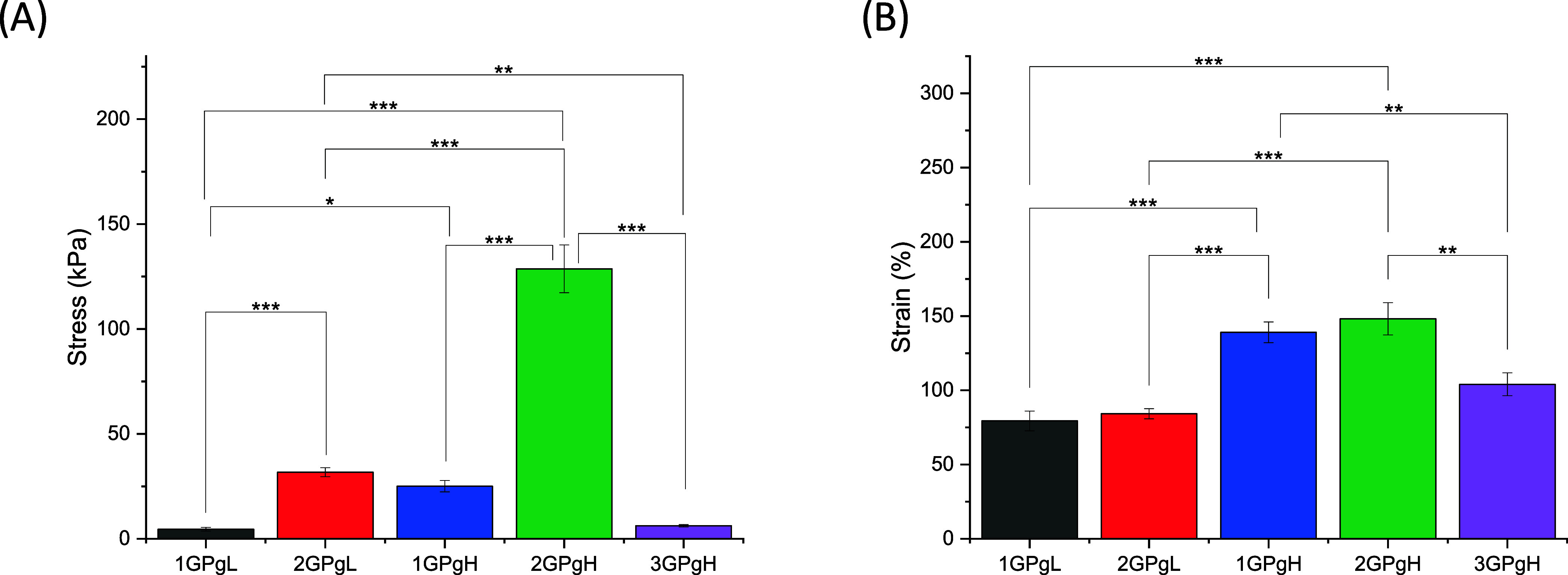
Comparison of applied stress on hydrogel films at a breakpoint (A) and an average elongation of hydrogel films at the breakpoint (B).

### 3.7 Hydrogel Films Morphology and Porous Structure

The self-improved A-ESEM microscope equipped with an ionization secondary electron detector with an electrostatic separator (ISEDS)^50^ was used to evaluate the hydrogel film morphology. Prior to the A-ESEM observation, all samples were prepared via freeze-drying of polymer blend solutions in various GK/PVA ratios. The initial freeze-drying process was essential to achieving the porous structure, and surface morphology was first visualized in the dry state (Figure 8). The hydrogel was then hydrated and in situ freeze-dried^51,43^ in a specimen chamber of the microscope. This rapid process allowed the stabilization of the inner porous structure to close to the natural state, as if applied as a transparent coating^52^ (Figure 9). To further assess the internal structure and the effect of the antiseptic coating, the sample was cracked after snapping in liquid nitrogen (Figure 11). In the dry state, the surfaces of samples were visualized as shown in Figure 8; sample 1GPgL (GK/PVA ratio of 1:1, 25 wt % glycerol) shows a clearly defined porous network, while sample 2GPgL (GK/PVA ratio of 1:2, 25 wt % glycerol) showed a smoother surface with a less distinct porous structure, likely due to a denser surface layer. Samples 1GPgH and 2GPgH (75% glycerol), as seen in Figure 8C,D, also displayed a less defined surface porosity attributed to a glycerol-rich polymer layer on the hydrogel surface. A-ESEM imaging revealed that these high-glycerol samples exhibit a sandwich-like structure, consisting of a smooth, dense outer layer and a porous internal network primarily composed of PVA, GK, and glycerol. This layered architecture enables the hydrogel to entrap and retain water within its structure, contributing to its functional performance. In addition, the presence of glycerol significantly influences the antifreezing properties,^53^ likely preventing ice crystal formation in the surface layer, which, combined with rapid freezing, inhibits pore development at the surface.^54^ This effect contributed to the formation of a compact, smooth outer layer, as also reported by Sornkamnerd et al.^55^ and Simoni et al.^56^

**Figure 8:**
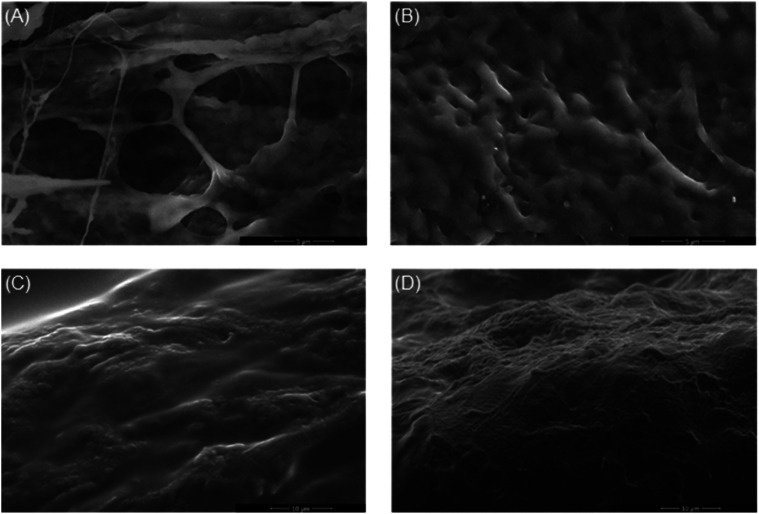
A-ESEM surface images of GK-based hydrogels after freeze-drying: (A) 1GPgL, (B) 2GPgL, (C) 1GPgH, and (D) 2GPgH.

**Figure 9:**
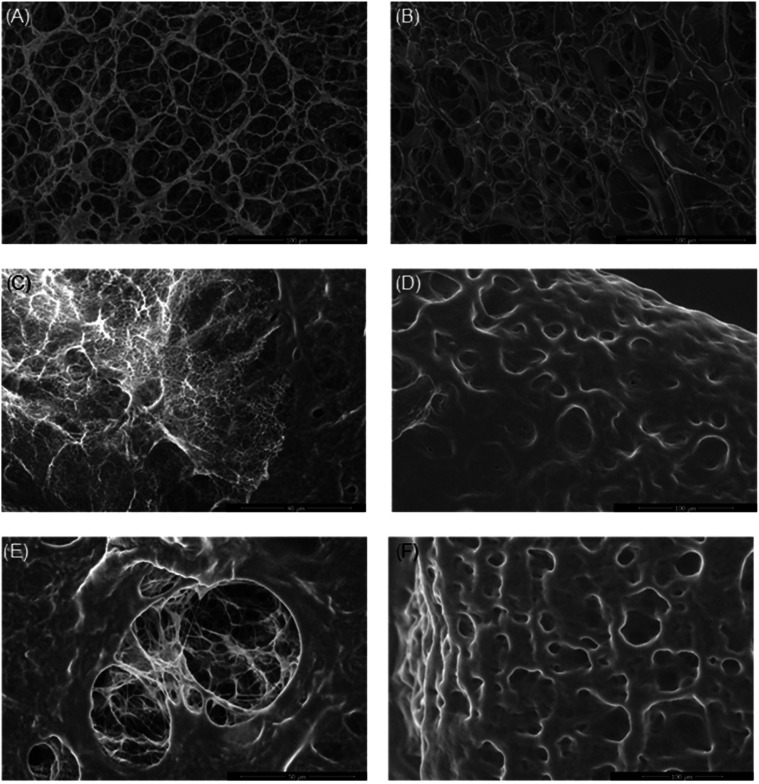
A-ESEM images after in situ freeze-dried hydrogels of 1GPgL (A), 2GPgL (B), 1GPgH porous network (C), 1GPgH hydrogel surface (D), 2GPgH porous network (E), and 2GPgH hydrogel surface (F).

After hydration and in situ freeze-drying, the internal morphology of the hydrogels became more apparent (Figure 8A,B). Samples after hydration and subsequent in situ freeze-drying tend to have rounder and smoother pore walls; Figure 9A,B. Series 1 samples (25% glycerol) exhibited a microporous network with average pore sizes of 7.8 ± 5.1 and 15.6 ± 13.6 μm for 1GPgL and 2GPgL, respectively (Figure 10). In contrast, Series 2 samples (75% glycerol) showed smaller average pore sizes: 5.4 ± 3.3 μm (1GPgH) and 3.8 ± 2.0 μm (2GPgH). This reduction in pore size is consistent with the formation of a dense surface layer and the plasticizing effect of glycerol, which limits ice crystal growth during freeze-drying.

**Figure 10:**
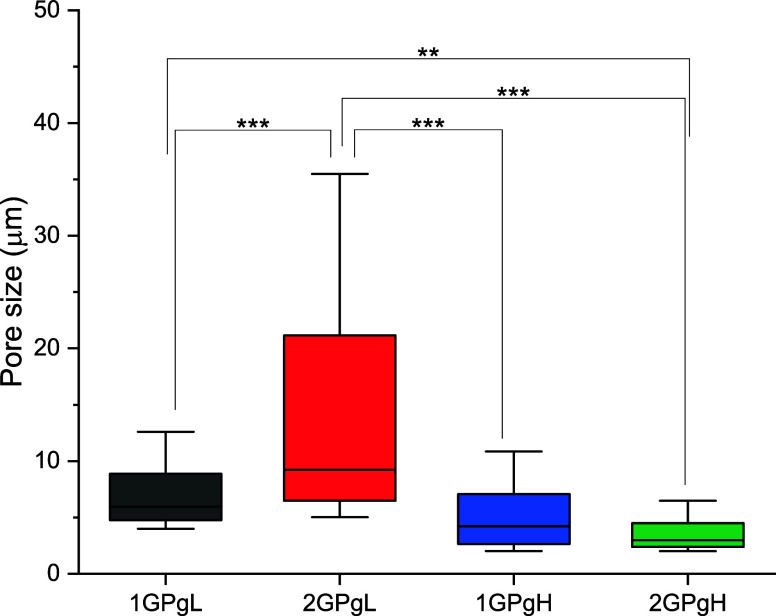
In situ freeze-dried hydrogel pore size from the A-ESEM image estimated by SEMIPE.

The visual differences between the surface layers before (Figure 8C,D) and after in situ freeze-drying (Figure 9D,F) further confirm that the second freeze-drying step alters the surface morphology. The chemical composition and polymer ratio strongly influence both the surface appearance and the internal porosity. Higher PVA content (e.g., in 2GPgL) results in thicker pore walls and slightly larger pores, while a higher GK content (e.g., in 1GPgL) leads to thinner walls and more uniform pores. In contrast, increasing glycerol concentration results in a smoother, denser outer layer and reduced pore size beneath the surface. Finally, samples 1GPgH and 2GPgH were coated with antiseptic OCT, and A-ESEM was used to visualize the morphological changes. In the dry state (Figure 11C,D), no visible antiseptic crystals or surface deposits were observed. Similarly, lateral cuts (Figure 11A,B) showed no distinct antiseptic layers. These findings suggest that the antiseptic was absorbed into the dense surface layer and possibly diffused into the hydrogel bulk. This absorption is likely facilitated by the ethanol-based OCT solution, which temporarily disrupts the glycerol-rich surface layer before it evaporates, resulting in a smooth, porous surface.

**Figure 11:**
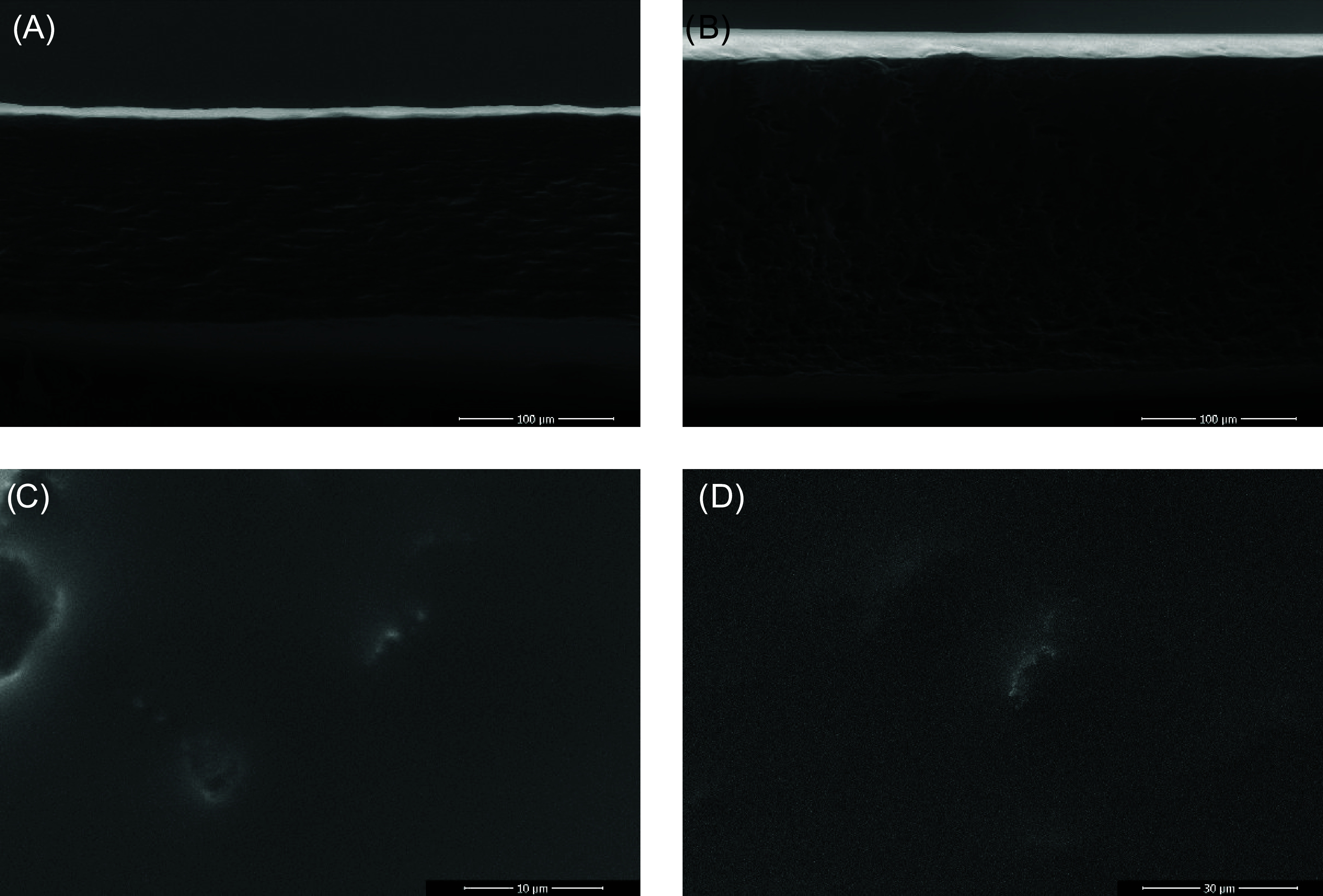
Lateral cuts through the nonhydrated samples 1GPgH-oH (A), 2GPgH-oH (B), in situ freeze-dried, and hydrogel surface of 1GPgH-oH (C) and 2GPgH-oH (D).

Because there is no visible antiseptic layer on the surface, energy-dispersive X-ray spectroscopy (EDS) analysis was performed on the coated hydrogels to verify and determine the presence of the OCT on the hydrogel film surface or in the matrix, focusing on the detection of chlorine (Cl) and nitrogen (N) elements. Table 3 presents the cross-sectional elemental analysis of samples 1GPgH and 2GPgH, both with and without the OCT coating. Although the EDS technique provides only approximate values, the results confirm the presence of OCT at low concentrations. Chlorine was detected at the detection limit in both coated samples: 1.29% in 1GPgH-oH and approximately 0.1% in 2GPgH-oH. The lower chlorine signal in 2GPgH-oH may be due to the dense glycerol-rich surface layer, which limits OCT penetration and thus reduces elemental detectability. In contrast, an increase in nitrogen content by 0.2% in 1GPgH-oH compared to the uncoated sample suggests a higher degree of OCT absorption. These findings indicate that the hydrogel morphology and surface structure significantly influence OCT penetration, with denser surface layers impeding diffusion into the matrix. Despite the low elemental concentrations detected, the amount of OCT present is considered sufficient for antibacterial activity, as discussed in Section 3.9. The exact concentration of the coated OCT was further confirmed by UV–vis analysis in the release study presented in Section 3.8.

### 3.8 Release Kinetics of Octenidine Dihydrochloride

A cumulative in vitro release study determined the release of the OCT from GK-based hydrogels. Given the nature of the sample and the fact that OCT is coated on the hydrogel surface, an initial rapid release of the drug was expected. The presence of OCT on the hydrogel surface was confirmed using EDS analysis in Section 3.7, despite the analysis reaching its low limit. Figure 12 shows a rapid OCT release followed by a sustained release over 2 weeks. The release kinetics are influenced by the coating method, hydrogel composition, and hydrogel film behavior in an aqueous environment, as hydrogel easily binds water, indicating that the release of the OCT might also be dependent on the swelling behavior and its subsequent hydrolytic stability, as described in Sections 3.2 and 3.3. Figure 12 shows that all release profiles have similar curves with the expected initial significant burst release of OCT, as described by Moritz et al.^33^ Curve profiles indicate that the release mechanism is consistent across all of the tested samples. To understand the release kinetics of the OCT from the hydrogel, release curves were fitted with Higuchi and KP mathematical release kinetics models. The KP model fitted the best for all tested samples with the correlation coefficient *R* ^2^ values over 0.97–0.99 and *n* exponent ≤0.46, indicating that Fickian diffusion contributes to OCT release from the hydrogel surface.

**Figure 12:**
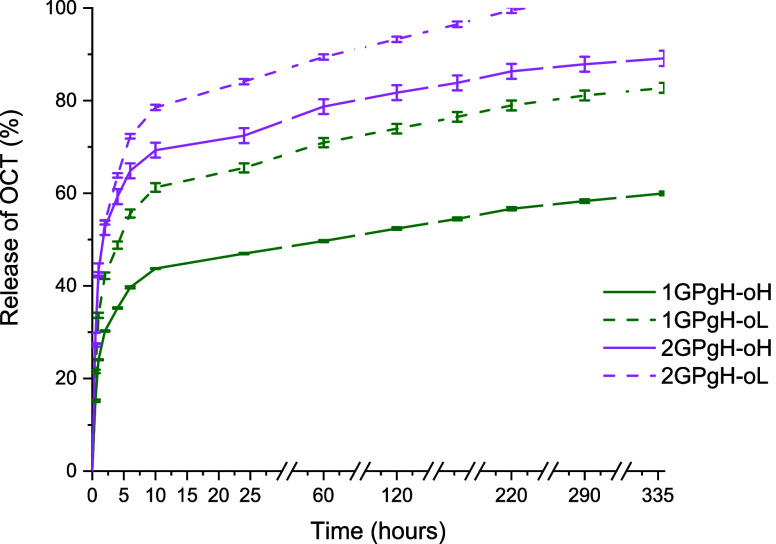
Cumulative release of the OCT from the hydrogel film surface.

Diffusion is driven by the concentration gradient between the OCT in the hydrogel’s surface layer and the solvent. The concentration gradient is highest at the beginning of the experiment, correlating with the measurement results. 24% OCT was released from sample 1GPgH-oH (having a higher concentration of 0.1 μg·cm^–2^) within the first hour, while 34% was released from sample 1GPgH-oL (0.05 μg·cm^–2^). Conversely, 2GPgH samples showed a faster release, approximately 42–43% in both samples (2GPgH-oL/H), indicating that binding of the OCT to the hydrogel surface and matrix beneath depends on the composition. This uncoated sample (2GPgH) also showed the highest stiffness and elongation, indicating a strong cross-linked network and thus releasing the highest amount of OCT that was probably weaker bound to the hydrogel film surface than in the case of 1GPgH-oL or 1GPgH-oH. After the initial burst, all hydrogel film release profiles became more gradual but continuous even after 14 days, confirming the diffusion mechanism driven by decreasing the OCT concentration over time. Only the 2GPgH-oL sample released all of the weakly coated OCT during the experiment. Generally, samples with a lower OCT concentration released approximately 10–20% higher amounts of OCT than those with a higher concentration, suggesting that OCT concentration influences binding to the hydrogel. A lower dose might not disrupt the hydrogel surface layer, allowing for easier diffusion when in contact with a solvent. As shown in Figure 12, OCT was present in samples 1GPgH-oL/H and 2GPgH-oH at the end of the experiment (after 14 days). This indicates that the OCT is both coated on the surface and also loaded deeper into the hydrogel matrix. The dense surface layer and OCT’s ability to load deeper under the surface layer into the hydrogel matrix might cause OCT entrapment, making OCT release slower.

#### 3.8.1 Cytotoxicity of OCT-Coated Hydrogel Films

The extract method was used to assess the cytotoxic effect of the hydrogel films on NIH-3T3 fibroblasts. This method was adopted for the in vitro cytotoxicity evaluation of materials that may release leachable toxic components from the material or exposed surface. The extraction conditions (time ≈ 24 h and temperature ≈ 37 °C) were chosen according to the physicochemical characteristics of the hydrogels, such as degradation and OCT release.Figure 13A and B show the viability of NIH-3T3 cells exposed to extracts of GPgL series with 25 wt % glycerol and GPgH series with 75 wt % glycerol. The extracts of the OCT-free sample did not induce any significant cytotoxic effect on cells compared with the plastic control. In fact, viability decreased the most (79%; *P* ≤ 0.01) for the 2GPgH hydrogel.

This could be attributed to the high glycerol release from the sample, according to the higher percentage of hydrogel degradation (32% after 24 h).^57^ Considering PVA as a biocompatible material, glycerol at higher concentrations has also shown cytotoxic effects, as reported elsewhere.^58,59^ The viability assay of the selected antiseptic extracts of the OCT sample is shown in Figure 13B. The assay revealed a dependence on cytotoxicity on the OCT concentration in NIH-3T3 fibroblasts, which is correlated with the OCT release test (Figure 12). In our results, the viability of the 1GPgh-oL sample is 60%, and the viability of 1GPgh-oH, 2GPgh-oL, and 2GPgh-oH samples reached less than 50%. These results correspond to the decreased viability of cells in the presence of OCT in samples in a dose-dependent manner, as reported elsewhere.^60,61^

**Figure 13:**
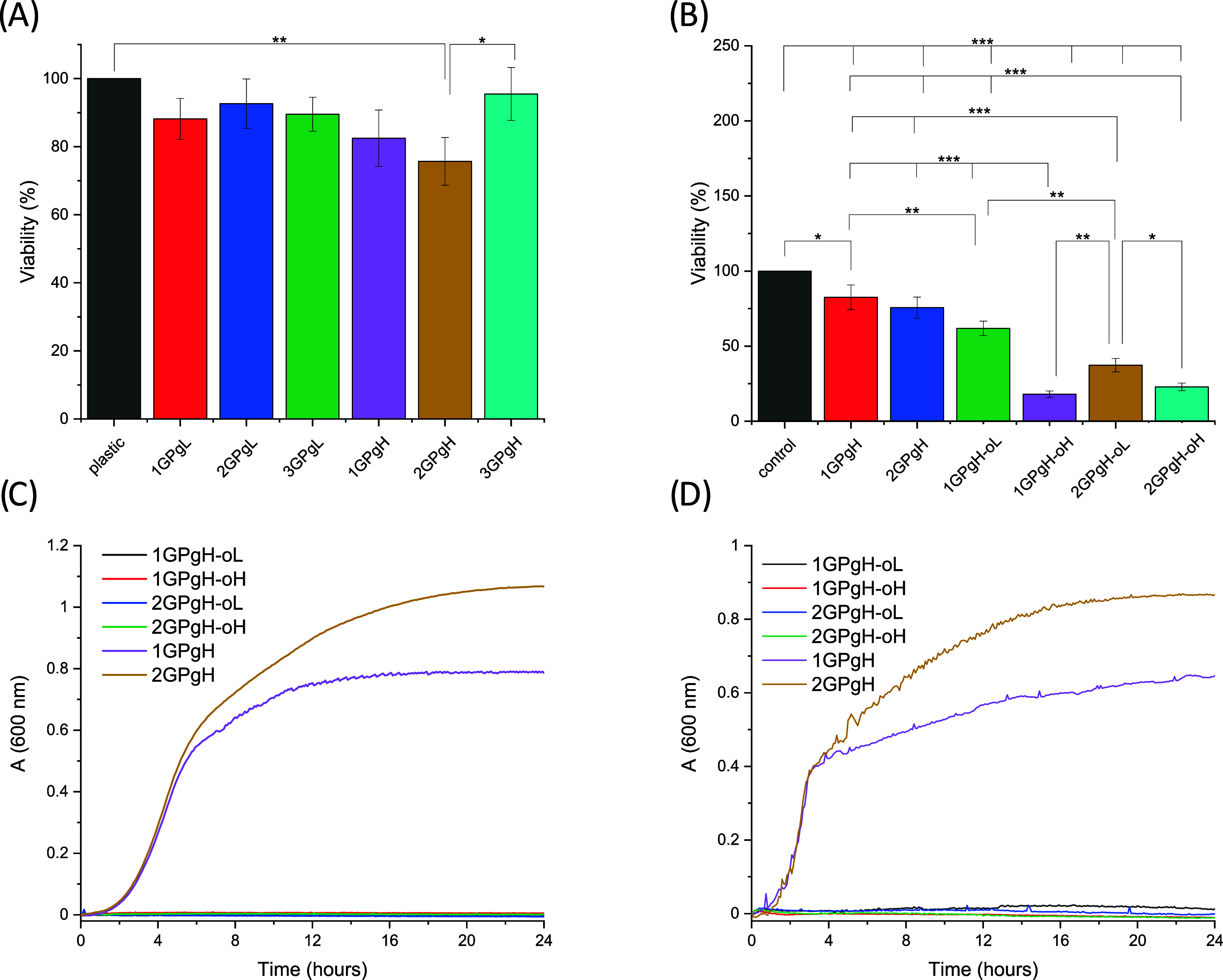
Cytotoxicity studies of OCT-free hydrogel extracts (A) and OCT-coated hydrogel films extracts (B); antibacterial activity of OCT-coated GK-based hydrogel films against Gram-positive and Gram-negative resistant bacterial strains: *S. aureus* (C) and *E. coli* (D).

### 3.9 Antibacterial Activity of OCT-Coated Hydrogel Films

Antibacterial activity against multidrug-resistant bacterial strains is an essential property nowadays, since it is a worldwide problem requiring innovative solutions for effectively suppressing and eliminating bacterial infections. Therefore, the GK-based hydrogel films 1GPgH and 2GPgH with and without coated OCT were tested for antibacterial activity using the broth microdilution methodology. The antimicrobial effect was determined based on the growth curves obtained from individual bacterial strains. *S. aureus* and *E. coli* were chosen to represent Gram-positive and Gram-negative bacterial strains. From the results shown in Figure 13C,D, we can conclude that materials without coated OCT (1GPgH and 2GPgH) do not significantly inhibit the growth of bacterial strains. The GK in the hydrogel matrix shows limited antibacterial properties since it has a low concentration. Anyhow, sample 1GPgH, having a higher amount of GK, exhibited much higher bacterial inhibition than 2GPgH, where PVA prevails. Drápalová et al.^48^ and Lipový et al.^62^ proved GK’s antibacterial activity on different bacterial strains. On the other hand, hydrogel films coated with OCT showed complete inhibition of bacterial growth in both tested materials and concentrations, indicating sufficient OCT elution from the hydrogel with the desired antimicrobial effect. Therefore, we can conclude that even low OCT concentrations (0.05 and 0.1%) exhibit high antibacterial efficacy compared to the literature.^63,64^

## 4 Conclusions

This study successfully developed and characterized innovative freeze-dried hydrogel films based on GK and PVA with antibacterial OCT, showcasing insight into the effect of composition and their potential for various applications beyond wound healing. The films demonstrated unique physicochemical properties, influenced by the specific composition and fabrication method, which shaped their distinctive morphology and overall performance. Enhanced transparency, mechanical stability, nontoxicity, and antibacterial efficacy were achieved by carefully manipulating GK, glycerol, and PVA content coated with OCT. Hydrogels with higher glycerol concentrations exhibited smaller pore sizes, leading to increased light transmission, thus improving transparency and enhanced elasticity with mechanical stability, making them suitable for transparent coatings and biomedical devices. The hydrogels maintained long-term stability in wet environments, as shown by swelling and degradation studies, particularly those with higher PVA and glycerol content. Adding low doses of OCT significantly improved antibacterial activity against Gram-positive (*S. aureus*) and Gram-negative (*E. coli*) bacteria with sustained release over several days, effectively suppressing infections. Biocompatibility assessments indicated that while GK/PVA hydrogels were not cytotoxic, incorporating OCT increased cytotoxicity, suggesting that optimizing OCT concentration could enhance cell viability while preserving antibacterial efficacy. These insights into the composition–structure relationship highlight the versatility of GK-based hydrogels as multifunctional materials, with potential applications extending from wound healing and infection control to other uses requiring transparent, mechanically stable, and antibacterial surfaces. According to the aforementioned requirements, the best composition fulfilling all goals seems to be the sandwich-like sample 1GPgH-oL involving 1:1 GK/PVA, 75% glycerol, and only 0.05% coated octenidine dihydrochloride. The sample is transparent in both dry and hydrated states, showing great porosity, elasticity and good stiffness, great stability, noncytotoxicity, and excellent antibacterial efficacy. Hydrogels have remarkable antibacterial efficacy against both Gram-positive and Gram-negative bacteria, specifically against *S. aureus* and *E. coli*, respectively. Future research will explore further enrichment with alternative antimicrobial agents, broadening the scope of applications of these innovative biomaterial hydrogel films. Their adaptability makes them suitable for drug delivery systems, tissue scaffolds, flexible bioelectronics, and environmental remediation, demonstrating their broad impact on various fields.

## Data Availability

Supporting data associated with this article can be found online as a Data set at Zenodo: 10.5281/zenodo.15738067.
